# Autism prevalence and the limits of diagnostic expansion: a perspective on diagnostic validity, adult assessment, and phenotypic stratification

**DOI:** 10.3389/fpsyt.2026.1917652

**Published:** 2026-07-22

**Authors:** Gabriela N. F. Guimarães

**Affiliations:** Department of Psychology, College of Liberal Arts, The University of Texas at Austin, Austin, TX, United States

**Keywords:** autism spectrum disorder, diagnostic expansion, heterogeneity, phenotypic stratification, psychiatric comorbidity

## Abstract

The reported prevalence of autism spectrum disorder (ASD) has risen dramatically over the past two decades. Although increased awareness, broader diagnostic criteria, and improved access to assessment have corrected historical under-identification, this diagnostic expansion also raises a significant methodological concern: the risk of diagnostic dilution. Increasingly, surveillance systems and clinical cohorts may include individuals whose phenotypic profiles, developmental histories, and functional impairments do not fully align with a developmentally anchored neurodevelopmental presentation of ASD. This challenge is particularly acute in adolescent and adult assessments, where developmental history may be incomplete and standardized instruments or self-report measures may show limited specificity when applied to clinically complex psychiatric populations. Conflating developmentally anchored ASD with partially overlapping clinical phenotypes may reduce the signal-to-noise ratio in genetic, biomarker, neuroimaging, and therapeutic research, contributing to findings that are difficult to replicate or interpret. To preserve diagnostic validity, this Perspective argues that best-estimate clinical diagnosis must be grounded in rigorous developmental anchoring, collateral information, and judicious clinical judgment. It further proposes a set of core stratification domains for systematic phenotypic stratification, including age at first concern and diagnosis, biological sex and sex-related ascertainment factors, language and cognitive trajectories, adaptive functioning, intellectual disability, psychiatric comorbidities, ascertainment source, diagnostic instruments used, collateral developmental documentation, and support needs and functional impairment across contexts and over time. Stratification should not be understood as a restriction on clinical access, but as a scientific requirement for meaningful prevalence estimates and biologically informative autism research.

## Introduction

Over the past two decades, the reported prevalence of autism spectrum disorder (ASD) has risen dramatically, from approximately 0.4–2 per 1,000 in the 1960s and 1970s to current estimates exceeding 1 in 32 children in the United States (1–3). This trajectory has been met with both justified concern and considerable methodological debate. There is broad agreement that improved awareness, expanded diagnostic criteria, and better access to services have corrected genuine historical under-ascertainment, particularly among females, verbally fluent individuals, and adults presenting for the first time (2, 4). Yet these welcome developments do not fully explain what is now being counted as autism, nor do they guarantee that what is being counted corresponds to the same underlying construct. The central argument of this Perspective is that the very success of diagnostic expansion now carries a risk of diagnostic dilution: the inclusion of individuals whose phenotypic profiles, developmental histories, and functional impairments may not align with the core neurodevelopmental construct that autism has historically described (5). This risk is especially acute in the assessment of adolescents and adults, where differential diagnosis is most challenging and where standardized instruments show limited specificity (6).

Concerns about diagnostic dilution are not tantamount to denying the validity of adult-diagnosed autism or of milder presentations, nor do they dismiss the real needs of late-identified individuals. Rather, they point to the scientific imperative of diagnostic validity, ensuring that prevalence estimates, biomarker studies, genetic research, and clinical trials rest on diagnostic valid, developmentally anchored, and well-characterized samples. The discussion that follows outlines the evidence for diagnostic expansion, the specific challenges of adult diagnosis, and a constructive research agenda centered on phenotypic stratification.

## Diagnostic expansion: evidence of accretion and substitution

Empirical work has demonstrated that changes in diagnostic practices account for a substantial fraction of rising prevalence. In a landmark retrospective record review of 7,003 clients enrolled with the California Department of Developmental Services, King and Bearman estimated that 26.4% of the increased autism caseload between 1992 and 2005 was uniquely attributable to diagnostic change, specifically, to diagnostic accretion (the addition of an autism diagnosis to a pre-existing diagnosis of intellectual disability) and diagnostic substitution (the replacement of an earlier diagnosis with autism) (1). The odds of an individual acquiring an autism diagnosis were significantly elevated in years coinciding with changes to DSM criteria and California-specific diagnostic guidelines (1). These findings have been corroborated by other studies: as Simonoff noted, broadening diagnostic boundaries, improved identification, diagnostic substitution, and service access all contribute to rising rates, although no evidence suggests that people without significant impairment are currently being diagnosed (4). Blenner and Augustyn emphasized that the ADDM network’s methodology, while useful for surveillance, is not designed to determine whether autism is truly increasing in the population, only that we are counting differently (2).

Fombonne offered a more trenchant methodological critique, highlighting that recent prevalence surveys incorporate screening tools with mediocre specificity, employ weighting assumptions that may inflate estimates, and rely on caseness definitions, particularly PDD-NOS, that are vulnerable to contamination by phenocopies (2). He noted that in a recent research sample, over 30% of participants referred with a pre-existing autism diagnosis did not have the diagnosis confirmed upon rigorous, videotaped assessment (2). Such findings do not imply that autism is over diagnosed in all settings, but they do indicate that the boundary between ASD and other developmental and psychiatric conditions has become porous, a problem that intensifies when cases are identified through record review alone, without direct clinical assessment.

The call for diagnostic validity should not be interpreted as gatekeeping or as a denial of support for individuals first assessed or formally identified later in life. Clinical services may appropriately respond to functional need even when etiological or diagnostic certainty is incomplete. Research classification, however, serves a different purpose. For prevalence estimation, biomarker discovery, genetic studies, and clinical trials, diagnostic categories must be sufficiently coherent to support inference. Thus, access to support and research stratification should be treated as related but non-identical goals.

## The challenge of diagnosing adolescents and adults

If diagnostic dilution is a concern, it is most acute in the assessment of adolescents and adults. Adult autism diagnosis presents unique difficulties: developmental history may be unavailable or unreliable; learned compensatory strategies and camouflaging can mask core symptoms; and high rates of co-occurring psychiatric conditions, anxiety, depression, ADHD, personality disorders, and psychotic disorders, create overlapping symptom profiles that challenge even experienced clinicians (7–10).

Late diagnosis should not be conflated with late onset. Camouflaging, compensation, and missed recognition may delay identification, particularly among females, verbally fluent individuals, and individuals from underserved communities. However, these factors do not remove the requirement that ASD be anchored in early neurodevelopment (11, 12). In adult assessment, the central question is not merely whether autism-like symptoms are currently endorsed, but whether a coherent developmental trajectory can be reconstructed from collateral history, school records, medical documentation, informant report, or other longitudinal evidence (13).

The psychometric limitations of existing instruments in adult populations are well documented. Wigham and colleagues conducted a systematic review of structured questionnaires and diagnostic measures for ASD in adults, concluding that sensitivity and specificity were best when comparing previously diagnosed ASD cases to general population controls, but were substantially reduced in clinic-referred samples (10). In mental health settings, the use of a single structured questionnaire was unlikely to accurately differentiate ASD from mental health conditions (10). Similarly, Conner and colleagues found that in an outpatient adult ASD clinic, the ADOS had only fair correspondence with clinician diagnosis (AUC = 0.69), while the RAADS-R (AUC = 0.58) and AQ (AUC = 0.40) performed even more poorly (9). Notably, AQ scores were higher in the non-ASD group than in the ASD group, raising serious questions about the use of self-report measures as diagnostic proxies (9).

Matson and Neal emphasized that the diagnostic instruments available for adults were largely developed and normed on child populations, and that claims of “gold standard” status for particular tools rest on extrapolation rather than direct testing in adult cohorts (7). Shulman and colleagues further underscored that the ADOS-2 and ADI-R, while robust in research settings with well-characterized samples, show reduced specificity when applied to individuals with co-occurring psychiatric disorders, particularly schizophrenia, personality disorders, and psychosis (8). They noted that in one study, 58% of false-positive cases on the ADOS-2 Module 4 had a diagnosis of personality disorder (8). These findings point to a critical gap: the instruments we rely upon were not designed or validated for the heterogeneous, psychiatrically complex populations now presenting for first-time diagnosis in adulthood.

## The risk of misclassification and its consequences

Diagnostic dilution has direct implications for research and clinical practice. If prevalence samples increasingly include individuals whose social communication difficulties arise from anxiety, trauma, ADHD, personality disorder, or intellectual disability rather than from a neurodevelopmental condition with early-onset, life-course characteristics, then prevalence estimates lose interpretive clarity. More concerning, however, is the impact on biomarker discovery, genetic association studies, and treatment trials. As Georgiades and colleagues argued, the failure to address phenotypic heterogeneity has hampered the field’s ability to identify replicable biological markers or predict treatment response (14). When study samples conflate true neurodevelopmental autism with phenocopies arising from diverse etiologies, the signal-to-noise ratio in neuroimaging, genomic, and pharmacological research is degraded, and the likelihood of false-negative or non-replicable findings increases.

Simonoff cautioned that heritability estimates of 90% leave little scope for environmental factors only if one ignores gene-environment interplay (4). But her central point, that etiological research requires careful phenotyping and measurement of both genetic and environmental factors, applies equally to the need for stratification. Foss-Feig and colleagues proposed a dimensional framework borrowing from the schizophrenia literature, clustering ASD features into positive (atypical behaviors present), negative (expected behaviors absent), and cognitive dimensions, arguing that such an approach could improve diagnostic precision and illuminate distinct neural and genetic pathways (15). This proposal aligns with the broader recognition that heterogeneity is not noise to be eliminated but information to be harnessed.

## Developmental anchoring and the primacy of clinical judgment

A recurring theme across the literature is that standardized tools and self-report measures cannot substitute for a thorough developmental history, collateral information, and informed clinical judgment. Shulman and colleagues emphasized that the first step in differential diagnosis is obtaining an extensive developmental history to examine the consistency of symptoms over time and their pervasiveness across contexts (8). When ASD is the primary diagnosis, core social communication challenges should frame the individual’s interactions from early childhood, and restricted, repetitive behaviors should be present from an early age, even if they evolve in form over development. The absence of such developmental anchoring raises the possibility that an alternative diagnosis better accounts for the presenting picture.

For adolescents and adults, this principle is especially critical. Individuals first assessed or formally identified later in life genuinely do exist, particularly women, verbally fluent individuals, and those from underserved communities whose early difficulties were overlooked or misattributed. But the validity of adult diagnosis depends on the ability to reconstruct, through whatever sources are available (parental report, school records, medical history, home videos), evidence of early-emerging socio-communicative differences (10, 12, 16). Without such evidence, the differential diagnosis widens to include social anxiety disorder, avoidant personality disorder, schizoid personality disorder, obsessive-compulsive disorder, ADHD, and trauma-related conditions, all of which can produce autism-like presentations in adulthood without representing autism per se.

This Perspective focuses on diagnostic and phenotypic validity at the point of sample construction. It does not argue that developmentally anchored ASD samples will be biologically homogeneous, nor that phenotypic stratification eliminates all within-ASD variance. Rather, diagnostic validity is a necessary precondition for the interpretable study of such variance. Once a sample has been anchored to a coherent neurodevelopmental construct, biological, developmental, and functional heterogeneity should be examined rather than averaged away. However, analyses of within-ASD heterogeneity are only meaningful when the sample first corresponds to the clinical-developmental construct under investigation. This distinction is important because diagnostic dilution and within-sample neurobiological heterogeneity represent related but non-identical threats to inference. The present framework addresses the former by strengthening population-level diagnostic validity; it does not claim to resolve the latter, which requires additional biological, developmental, genetic, metabolic, and sex-informed stratification within diagnostically anchored ASD cohorts.

## A research agenda for phenotypic stratification

To preserve diagnostic validity while maintaining the hard-won gains in recognition and access, the field requires a systematic program of phenotypic stratification. Specifically, future research should:

1. Stratify by age at first concern and age at diagnosis. Participants diagnosed in early childhood, mid-childhood, adolescence, and adulthood likely represent different populations with different etiological contributions, developmental trajectories, and support needs. These subgroups should be analyzed separately rather than pooled.
2. Stratify by biological sex and report sex-related ascertainment factors. Biological sex should be reported not only as a demographic variable, but also as a potential modifier of age at recognition, symptom expression, diagnostic instrument performance, psychiatric comorbidity profiles, adaptive functioning, and support needs. However, sex-related differences in presentation or compensatory strategies should not be used to bypass the requirement for evidence of early developmental onset. In a neurodevelopmental condition, later formal identification must be distinguished from late onset; adult-ascertained cases should still require reconstructable evidence of early-emerging developmental differences.
3. Document language history and current language level. Language delay or deviance remains one of the strongest discriminators between ASD and other conditions. Studies should routinely report whether language milestones were met on time, whether language regression occurred, and current expressive and receptive language functioning.
4. Report cognitive and adaptive functioning profiles. Full-scale IQ, verbal-performance discrepancies, and adaptive behavior scores (e.g., Vineland) are essential for characterizing samples. Stratification by IQ range (e.g., 85) is a minimum requirement.
5. Track presence or absence of intellectual disability separately from ASD, and report co-occurrence rates transparently. The proportion of cases with and without ID has shifted dramatically over time and varies across studies, directly affecting comparability.
6. Record source of ascertainment. Whether participants are recruited from population screening, clinical referral, school records, or convenience samples has a major impact on sample characteristics. Ascertainment source should be reported and modelled as a covariate.
7. Assess and report psychiatric comorbidities systematically. Given the high rates of co-occurring conditions and their potential to mimic ASD, studies should include standardized measures of anxiety, depression, ADHD, and psychotic symptoms, and should report how differential diagnosis was handled.
8. Document the basis of clinician judgment. Best-estimate clinical diagnosis should remain the reference standard, but researchers should report which instruments, informants, and data sources contributed to that judgment, and whether diagnostic consensus was reached independently.
9. Incorporate collateral documentation whenever possible. School records, medical records, and informant reports (parent, partner, or long-term acquaintance) provide essential developmental context that self-report alone cannot supply.
10. Characterize support needs and functional impairment. The DSM-5 severity specifiers provide a starting point, but more granular measures of service utilization, adaptive functioning, and quality of life are needed. Impairment should be described across contexts and over time, not only in isolated situations or clinical encounters.
11. Report the proportion of participants meeting vs. not meeting the age-of-onset criterion (symptoms present in early developmental period), and analyses outcomes separately for these groups.

Such stratification would allow the field to distinguish between autism identified in early childhood with clear developmental delays, language difficulties, intellectual disability, or early functional impairment, and adult-ascertained presentations with retrospectively demonstrable early developmental features. Conflating these groups without adequate developmental documentation obscures etiological signals, inflates heterogeneity, and undermines the replicability of research findings.

### Minimum requirements versus ideal characterization

At minimum, research cohorts should report developmental onset evidence, age at first concern and diagnosis, language history, cognitive and adaptive functioning, intellectual disability status, psychiatric differential diagnosis, sex, ascertainment source, instruments/informants used, and functional impairment/support needs. Ideally, studies should additionally include collateral records, longitudinal developmental documentation, standardized comorbidity assessment, consensus diagnosis, and subgroup-specific or covariate-adjusted analyses.

Beyond reporting these variables descriptively, future ASD research should use them analytically. Studies should avoid relying exclusively on pooled group means and should, where sample size and design permit, examine subgroup-specific effects, covariate-adjusted models, variance partitioning by clinically meaningful dimensions, individual-level patterns, and heterogeneity of treatment, biomarker, genetic, or neuroimaging effects across sex, cognitive profile, adaptive functioning, support needs, and developmental trajectory.

## Conclusions

The rising prevalence of autism reflects a genuine public health success in recognizing a previously under-identified population. Yet diagnostic expansion has consequences. If prevalence becomes a moving target defined as much by service availability, diagnostic substitution, and screening methodology as by the underlying neurodevelopmental condition, the scientific foundation of autism research risks being built on shifting sand. The solution is not to restrict access to diagnosis, many individuals identified later in life benefit meaningfully from identification and support, but to insist on rigorous, developmentally anchored, multi-method assessment, and to stratify research samples with the granularity that the heterogeneity of the spectrum demands. Only then can prevalence be interpreted meaningfully, biomarkers identified robustly, and treatments matched to the specific needs of the individuals they are designed to serve.

## Data Availability

The original contributions presented in the study are included in the article/supplementary material. Further inquiries can be directed to the corresponding author.
